# The crux of decisions about who requires high-security care: A systematic analysis of referrals to the State Hospital

**DOI:** 10.1177/00258024251362542

**Published:** 2025-07-25

**Authors:** Lindsey Gilling, Duncan Alcock, Lindsay Thomson

**Affiliations:** 1 Division of Psychiatry, University of Edinburgh, Edinburgh, UK; 2 2294 Medical Department, The State Hospitals Board for Scotland, Carstairs, UK

**Keywords:** Forensic psychiatry, high-security, referral, assessment, admission, regression

## Abstract

There has been limited study to date into the specific patient factors that influence decisions about an individual's need for high-security psychiatric care. Admission to high-security services requires careful assessment and consideration to ensure patients receive the least restrictive care justified, and those who are most likely to benefit from it are admitted. This retrospective case-control study describes the demographic, clinical and risk characteristics of referrals made to the State Hospital in Scotland during a 12-month period, and delineates differences between referrals that were accepted and rejected for admission. It updates the methods of a previous study undertaken at the State Hospital. Six variables differentiated rejected and accepted referrals in univariate analyses. Multivariate logistic regression found that a model with only the individual's age, whether they were prescribed antipsychotic medication at the time of referral, and whether they had a history of violent convictions, best predicted referral outcome. The findings support the conclusion that it is not only an individual's risk of violence or the severity of their mental illness in isolation, but the combination of these factors that is deemed to necessitate high-security care within a stratified forensic mental health system.

In the United Kingdom, detention in high-security hospitals is reserved for people with mental disorder (including major mental illness, personality disorder, and intellectual disability), who often have a history of serious violence, and who pose a risk of serious harm to the public. The State Hospital at Carstairs is one of four high-security hospitals in the United Kingdom, serving all of Scotland and Northern Ireland. The hospital cares for male patients with mental disorder or intellectual disability who are detained under the Mental Health (Care and Treatment) (Scotland) Act 2003 as amended by the Criminal Procedure (Scotland) Act 1995. The State Hospital's patient population has been well described and researched over the years, providing insights into the characteristics and needs of those requiring high-security care, as well as their long-term recovery outcomes.^1–5^

The conditions of security provided by the State Hospital have shifted as a result of the decentralisation of forensic mental health care in recent decades. Until the late 1990s, secure care did exist in some local hospitals in Scotland, but all care at a greater level of security was provided within the State Hospital.^4,6^ The hospital provided conditions of ‘special security’, which encompassed what is now referred to as medium and high-security care. A systematic review of patients at the State Hospital between 1992 and 1994 found that a majority (53.3%) did not require high-security care.^4^ Changes to mental health policy^7^ and legislation (introduction of the Mental Health (Care and Treatment) (Scotland) Act 2003) necessitated the development of medium and low-security units in order to provide patients with the least restrictive care they required. In the intervening years, three regional medium-security units opened (Orchard Clinic serving the East (2001), Rowanbank Clinic serving the West (2007) and Rohallion Clinic serving the North (2012)), as well as various local low-secure units. A Matrix of Security was developed describing aspects of physical and procedural security provided by the three security tiers and admission guidelines for the three tiers are clearly defined (see Table 1).^8,9^ With the opening of new regional and local units, and with the introduction of the right to appeal against being held in excessive security introduced initially for high-security patients,^11^ the State Hospital patient population reduced from approximately 240 to 120.

**Table 1.** Admission guidelines for high, medium, and low-security care in Scotland (adapted from the State Hospital Referral Policy and Procedures^11^).

Admission Guidelines	Low Secure	Medium Secure	High Secure
Graveness of violence (according to Kennedy^10^)	Grade 3 (e.g., repetitive assaults causing bruising; self-harm) Public order/nuisance offending	Grade 2 (e.g., use of weapons to injure; arson; sexual assaults; stalking with threats to kill)	Grade 1 (e.g., homicide; stabbing penetrates body cavity; skull fractures; strangulation; serial penetrative sexual assaults; kidnap; torture; poisoning)
Immediacy	Acute illness or crisis likely to resolve in 3-6 months	Relapses abrupt Unpredictable	Unpredictable Inaccessible to staff
Specialist forensic need	Recall or crisis of former medium/high-security patient	Arson	Sadistic paraphilias associated with violence
		Jealousy	
		Resentful stalking	
	Current mental state associated with violence	Exceeds low secure capacity	Exceeds medium security
Absconding	Impulsive absconding	Pre-sentence with serious charge	Can coordinate outside help
		Other obvious motivations to abscond	Past absconding from medium or high security
Public confidence issues	Short-term family sensitivities	Predictable potential victims	National notoriety
		Local notoriety	

Referrals to the State Hospital have been described previously,^2^ before the aforementioned changes to the Scottish forensic estate. Pimm et al.^2^ described State Hospital referrals received throughout the year 1999, and explored differences across multiple domains using a case-control analysis between referrals that were accepted and rejected. The study identified variables associated with admission to special security: previous admission to the State Hospital, a history of previous charges or convictions, psychotic features at assessment, a family history of mental disorder, and evidence that psychosis may have been involved in the individual's index offence or behaviours leading to referral. There were also a several variables associated with the referral being rejected, though Pimm et al.^2^ reasoned these likely reflected specifics of the referral and assessment process in place at the time, which have since changed. Changes to the State Hospital admissions policy^9,12^ have brought it more in line with the other UK high-security hospitals.^13,14^ Consultants can no longer admit patients directly except in an emergency; referrals are discussed at the hospital's weekly Patient Pathway Meeting (PPM), and assessments are multi-disciplinary, whenever possible. Referrals for admission are now clearly differentiated from requests for psychiatric opinions, a limitation of the Pimm et al.^2^ analyses. Excluding these likely data artefacts in Pimm et al.^2^ findings, an analysis of referrals to Rampton High Security Hospital over a 12-month period between 1994 and 1995 identified similar discriminating factors between accepted and rejected referrals.^15^ Berry et al.^15^ reported that admission to high security was associated with having a more severe mental illness, and a history of serious offences, including serious violence.

A handful of other studies have analysed referrals to high-security care, though through a more limited scope. Three studies examined process factors, for example, waiting times^13,16^ or agreement between assessing psychiatrist recommendations and admission panel decisions.^17^ Two studies have specifically considered the security needs of high-security referrals to Broadmoor High Secure Hospital.^14,18^ Brown and Lloyd^18^ prospectively rated OPRISK, a structured checklist of operationalised risk factors, for consecutive referrals to Broadmoor over an 18-month period. Similarly, Williams et al.^14^ reported a retrospective cohort study comparing DUNDRUM-1 and DUNDRUM-2 scales on accepted and rejected referrals over a two-year period. Both studies found higher security needs among referrals accepted for admission to high security at Broadmoor Hospital than among rejected referrals.

To the authors’ knowledge, only two studies have compared accepted and rejected high-security referrals on a range of patient domains.^2,15^ Admission to high-security services requires careful assessment and consideration, to abide by patients’ right to receive the least restrictive care necessary, and to effectively manage the scarce resource that is high-security care.^14^ An understanding of how patients who are accepted for high-security psychiatric care differ from those who are not accepted is warranted, particularly in terms of a range of relevant domains. As the State Hospital now provides high-security care within a robust forensic estate that clearly delineates high, medium and low-secure care, an analysis of recent referrals would offer generalisability to other high-security units. This study served to update the findings of Pimm et al.,^2^ examining the demographic, clinical and forensic characteristics of referrals to the State Hospital and delineate differences between referrals that are rejected and accepted for admission.

## Methods

### The State Hospital referral and assessment process

**Figure 1.**
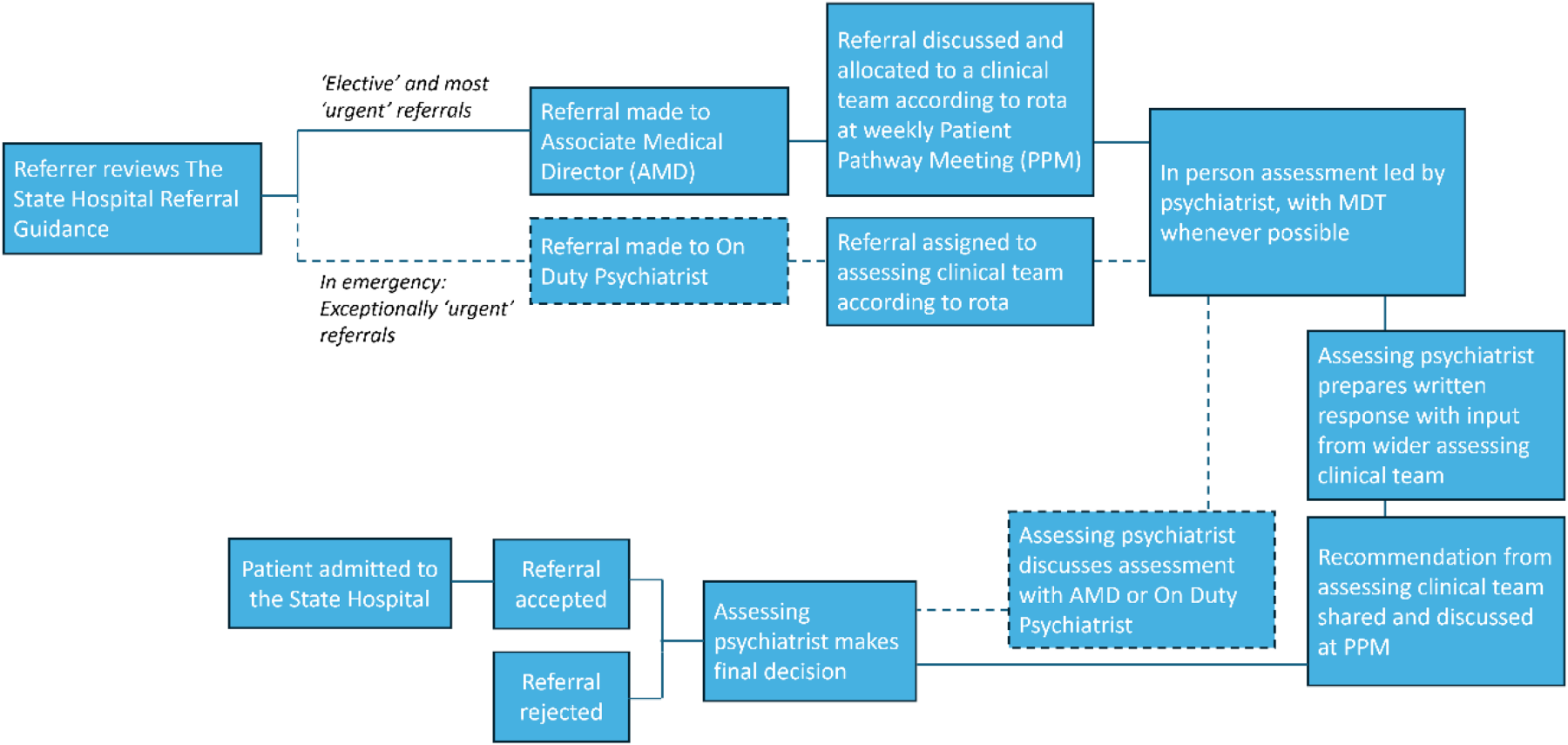
The State Hospital referral for admission process.

The State Hospital process for referrals and assessment is summarised in Figure 1. Referrals to the State Hospital (TSH) are made in writing to the Associate Medical Director, or, in an emergency, to the On Call Duty Consultant Psychiatrist. Referrals must provide sufficient information to allow for a decision to be reached as to whether the referral requires an assessment for high security. Referrals are brought to the PPM and discussed. The PPM is a multi-disciplinary meeting attended by all consultant psychiatrists with representation from psychology, nursing, allied health professionals, and social work. A decision is made at the PPM in the first instance to allocate to a multidisciplinary clinical team for assessment. The assessment is led by a consultant psychiatrist, or a specialist registrar acting on their behalf, joined by colleagues from other disciplines whenever possible and as relevant to the referred individual's specific needs. All professionals involved provide their assessment to the consultant psychiatrist leading the assessment. A recommendation from the assessing team is provided to the PPM. The PPM considers and discusses the recommendation and provides advice to the assessing team. The final decision to admit a patient to the hospital rests with the assessing consultant psychiatrist. This process can be agile for exceptionally urgent cases, through discussion with the Associate Medical Director or On Call Duty Consultant and can result in an assessment and even an admission taking place outside of the PPM.

### Study design

The study was a retrospective case-control analysis of referrals for admission to TSH received from 1 January to 31 December 2017. The sample was identified by reviewing the formal minutes of the PPM.^1^ Referrals received during 2017 that concluded in 2018 were also included. Not all referrals made during this time were for admission to high security. In rare instances, patients accepted by a medium-secure service, but where there is no bed available in medium security in Scotland to admit to, can be admitted to the State Hospital until a bed in medium security becomes available through the use of what is referred to as the ‘Exceptional Circumstances’ clause.^9^ These Exceptional Circumstances referrals are included in the summary below describing the referral process but excluded from all referral comparison analyses.

### Data collection

Data collection sources included PPM formal minutes and hospital electronic patient records. The hospital electronic patient records system was searched for all available reports and documentation provided to or produced by the State Hospital clinical team assigned to undertake the assessment for admission. These hospital records are held regardless of referral outcome. Data were recorded by a researcher into a standardised proforma. The proforma was developed with consideration to the variables measured by Pimm et al.,^2^ a previous data collection tool used to survey TSH patients,^4^ and knowledge of the information provided by practitioners in referral and assessment letters regarding the need for high-security care. A range of information was recorded across four major domains: relating to the referral process, demographic variables, clinical variables, and risk variables. A copy of the proforma is available upon request to the authors.

### Data analysis

To facilitate a comparison of findings, the analysis approach broadly followed that employed by Pimm et al.^2^ The data were entered into SPSS version 29^19^ and checked for data entry errors and missing values. Descriptive statistics including median (mdn) and interquartile range (IQR), were used to summarise referral sample characteristics and the referral process timescales. Referrals to high security were categorised as admitted (case) and rejected (controls) referrals according to the recorded decision at the PPM. Referrals made under the Exceptional Circumstances clause, or referrals made for high-security care but accepted for admission as medium security under the clause, were excluded from case-control comparisons, as were withdrawn referrals. Differences between cases and controls were explored first using univariate binary logistic regression. Odds ratios and corresponding 95% confidence intervals (CI) were calculated to provide effect size estimates.

In a second step, multivariate binary logistic regression was conducted with the variables shown to have a significant association with referral outcome in the univariate analyses. This step controls for potentially confounding relationships between the predictor variables. Backward elimination was used, which is a model selection technique that yields the most parsimonious model by removing variables one by one, using the Wald Test as the criterion to determine removal. The final model was evaluated using the Hosmer and Lemeshow Goodness of Fit test and by examining the proportion of variance in referral outcome explained by the predictor variables^20^ (Nagelkerke pseudo-R^2^).

## Results

### Referral process and outcomes

During the calendar year 2017 there were 55 referrals made to TSH, for 50 unique patients. Five patients were referred twice during that period. Seventeen referrals (30.91%) were for individuals who had previously been admitted to TSH. Forty-seven patients (85.45%) were referred to the major mental illness (MMI) service and six (10.91%) to the intellectual disability (ID) service. There was very limited information on two of the referrals, and it was not stated to which service the referrals were directed. Six referrals (10.91%) were made for medium-security patients under the Exceptional Circumstances clause.

The reason(s) for referral was/were noted for all but one case (1.82%). Most frequently, individuals were referred for reasons relating to assessing and treating the individual's symptoms of mental illness (n = 31, 56.36%), to managing harmful behaviours or absconding attempts (n = 23, 41.82%), to risks relating to their index offence (n = 23, 41.82%), and/or other reasons (n = 3, 5.45%). The referral was designated by the referring institution as ‘urgent’ in 21 cases (38.18%), and ‘elective’ for 32 cases (58.18%; urgency not known for 2 cases).

Of the 55 referrals, 47 were assessed by TSH (85.45%). Cases where an assessment did not occur included instances where the referral was withdrawn quickly by the referring institution (n = 3; 5.45%), and five instances (9.09%) where the referral was declined outright by the On Call Duty Psychiatrist due to being obviously inappropriate for high security. Assessments commenced a median of eight days (IQR = 13.00, n = 37) after referrals were made. The date the referral was made was unknown for 11 referrals (20.00%), which included several referrals via phone.

In this sample, 18 (38.30%) assessments were undertaken with just one discipline (psychiatry), 12 (25.53%) involved two and 12 (25.53%) involved three disciplines, and five assessments (10.64%) involved four or more disciplines. Psychology (n = 21, 44.68%) followed by nursing (n = 19, 40.43%) were most commonly involved alongside psychiatry. Referral outcome was reached a median of five days (IQR = 6.25, n = 44) following the assessment commencement, and 12.50 days (IQR = 15.75, n = 42) following receipt of referral. Following assessment, of the five cases referred under the exceptional circumstances clause (medium security patients), two were accepted (40.00%) and three rejected (60.00%). Following assessment, 32 individuals referred for high-security care were accepted (32 out of 47, 68.09%). Two of these individuals (6.25%) were initially referred for high-security care but, following the outcome of assessment, accepted for medium-security care under the Exceptional Circumstances clause. Therefore, excluding referrals that were withdrawn, the acceptance rate for high-security care was 66.67% (30 out of 45 referrals). The median number of days between date of referral and date of admission was 13.5 (IQR = 22.50, n = 30). Accepted patients were admitted a median of three days (IQR 6.50, n = 34) following referral outcome. The overall process was much faster for referrals considered urgent (Of all referrals, days from referral to referral outcome: Urgent referrals, Mdn = 6.00, IQR = 6.00, n = 21; Elective referrals, Mdn = 19.00, IQR = 19.00, n = 32. Admitted patients, days from referral to admission: Urgent referrals, Mdn = 7.00, IQR = 11.00, n = 15; Elective referrals, Mdn = 26.00, IQR = 23.00, n = 15).

### High-security referral characteristics

Characteristics of the high-security referrals were examined using descriptive statistics, with the number of missing cases for each variable reported in text or in Table 2. The referrals were all male, median age of 31.00 years (IQR = 13.00), and nearly all white (n = 38, 95.00%; missing n = 5) and British (n = 40, 93.00%; missing n = 2). Most were in a secure setting at the time of referral, either prison (n = 26, 57.78%) or medium or low-secure care (n = 12, 26.67%). Consultant psychiatrists led assessment in all but four cases (n = 9.3%), where a specialist registrar acted on their behalf. Many individuals had received one or more psychiatric diagnoses by the time of referral; most frequently this was schizophrenia or schizoaffective disorder (n = 21, 47.73%) or a substance use disorder (n = 12, 27.27%; diagnosis info missing n = 1). Thirty-nine individuals (88.64%) were noted to present with psychotic symptoms at the time of referral, regardless of whether a diagnosis had been made. A majority (n = 26, 61.90%) had previously received psychiatric inpatient treatment. Recent use of substances was not uncommon (n = 12; 29.27%) despite the majority of individuals referred being managed in secure settings. A large majority of referred individuals were already prescribed antipsychotic medication (n = 33, 80.49%) and eight (18.60%) were noted to be refusing medication. Most had forensic histories; 35 (85.37%) had prior convictions and 28 (68.29%) had prior violent convictions. Forty-one individuals referred (91.11%) were noted to have current charges or convictions, in all but two cases (95.1%) these were for violent offences. Eight patients (17.78%) had committed or were alleged to have committed murder or culpable homicide and four (8.89%) had committed or were alleged to have committed sexual offences. Weapons use in these index offences was very common (n = 26; 78.79%), frequently this was a knife or other bladed implement. Six individuals (13.95%) had absconded or attempted to abscond in their current setting.

**Table 2.** Variables influencing outcome of referral to high-security care. Univariate odds ratios (OR) and 95% confidence intervals (CI) in 45 referrals.

Variable	N	All patients	Rejected	Accepted	OR	95% CI	*p*-value
		N (%) or Mdn (IQR)	N (%) or Mdn (IQR)	N (%) or Mdn (IQR)			
*Process variables*							
Is currently in prison (ref: no)	**45**	**26** (**57.78)**	**5** (**33.33)**	**21** (**70.00)**	**4**.**67**	**1.24**–**17.60**	.**02**
Is currently in secure unit (ref: no)	45	12 (26.67)	6 (40.00)	6 (20.00)	0.38	0.10–1.47	.16
Is currently in other type of setting (ref: no)	45	7 (15.56)	4 (26.67)	3 (10.00)	0.31	0.06–1.60	.16
Referral noted as ‘urgent’ (ref: elective)	43	15 (34.88)	4 (26.67)	11 (39.29)	1.78	0.45–7.02	.41
Referred for treatment pathway ID (ref: MMI)	44	4 (9.09)	2 (14.29)	2 (6.67)	0.43	0.05–3.41	.42
*Assessment was led by specialist registrar (ref: led by consultant psychiatrist)	43	4 (9.30)	1 (7.69)	3 (10.00)	1.33	0.13–14.17	.81
Number of disciplines involved in assessment (continuous)	43	2.00 (2.00)	3.00 (1.50)	2.00 (2.00)	0.65	0.35–1.21	.17
Days to conclude referral (continuous)	37	14.00 (16.00)	19.00 (16.00)	12.00 (17.00)	0.97	0.92–1.02	.22
*Demographic variables*							
Age (continuous)	**44**	**31.00** (**13.00)**	**26.00** (**11.00)**	**31.50** (**12.00)**	**1**.**12**	**1.02**–**1.24**	.**02**
Was employed (prior to current detention) (ref: unemployed)	37	4 (10.81)	1 (8.33)	3 (12.00)	1.50	0.14–16.14	.74
Has educational or vocational qualifications (ref: no qualifications)	40	10 (25.00)	1 (7.69)	9 (33.33)	6.00	0.67–53.68	.11
*Has a family history of mental illness (ref: no)	32	17 (53.13)	7 (70.00)	10 (45.45)	0.36	0.07–1.75	.21
*Clinical variables*							
Was previously referred to TSH (ref: no)	45	18 (40.00)	5 (33.33)	13 (43.33)	1.53	0.42–5.58	.52
*Was previously admitted to TSH (ref: no)	45	14 (31.11)	3 (20.00)	11 (36.67)	2.32	0.52–10.04	.26
Was referred for assessment/ treatment of mental health (ref: no)	**45**	**28** (**62.22)**	**6** (**40.00)**	**22** (**73.33)**	**4**.**13**	**1.11**–**15.32**	.**03**
Received previous psychiatric inpatient treatment (ref: no)	42	26 (61.90)	8 (61.54)	18 (62.07)	1.02	0.27–3.93	.97
*Presents with psychotic symptoms (ref: no)	44	39 (88.64)	11 (78.57)	28 (93.33)	3.82	0.56–26.05	.17
Is currently prescribed antipsychotic medication (ref: no)	**41**	**33** (**80.49)**	**6** (**54.45)**	**27** (**90.00)**	**7**.**50**	**1.39**–**40.35**	.**02**
Is refusing medication (ref: no)	43	8 (18.60)	1 (7.69)	7 (23.33)	3.652	0.40–33.24	.25
Has historical alcohol problems noted (ref: no)	40	29 (72.50)	8 (66.67)	21 (75.00)	1.50	0.34–6.55	.59
Has recent drug use noted (ref: no)	41	12 (29.27)	5 (38.46)	7 (25.00)	0.53	0.13–2.18	.38
Has a history of trauma (ref: no)	33	12 (36.36)	5 (41.67)	7 (33.33)	0.70	0.16–3.02	.63
Has a history of self-harm (ref: no)	**36**	**21** (**58.33)**	**10** (**90.91)**	**11** (**44.00)**	**0**.**08**	**0.01**–**0.71**	.**02**
*Risk variables*							
*Is currently on remand (ref: no)	45	7 (15.56)	3 (20.00)	4 (13.33)	0.62	0.12–3.19	.56
*Is currently not in prison or legally detained (ref: detained)	45	3 (6.67)	2 (13.33)	1 (3.33)	0.22	0.02–2.70	.24
Physical violence led to referral – either referred due to index offence or managing physical behaviour (ref: no)	45	33 (73.33)	12 (80.00)	21 (70.00)	0.58	0.13–2.58	.48
Was referred for index offence (ref: no)	45	22 (48.89)	8 (53.33)	14 (46.67)	0.77	0.22–2.65	.67
Was referred for managing behaviour (ref: no)	45	17 (37.78)	7 (46.67)	10 (33.33)	0.57	0.16–2.03	.39
*Has previous convictions (ref: no)	41	35 (85.37)	10 (76.92)	25 (89.29)	2.50	0.43–14.54	.31
Has previous violent convictions (ref: no)	**41**	**28** (**68.29)**	**6** (**46.15)**	**22** (**78.57)**	**4**.**28**	**1.04**–**17.62**	.**04**
Has been violent during current detention (other than that leading to referral) (ref: no)	42	23 (54.76)	9 (75.00)	14 (46.67)	0.29	0.07–1.30	.11
Is convicted (ref: no)	40	26 (65.00)	7 (53.85)	19 (70.37)	2.04	0.52–8.00	.31
Years since index offence occurred (continuous)	33	3.60 (5.92)	3.44 (5.13)	3.81 (6.17)	1.01	0.87–1.18	.88
*Evidence that index offence may have been driven by psychotic symptoms (ref: no evidence indicating this)	27	8 (29.63)	2 (40.00)	6 (27.27)	0.56	0.08–4.24	.58
Used weapons in index offence (ref: no)	33	26 (78.79)	7 (77.78)	19 (79.17)	1.09	0.17–6.94	.93
Attempted to abscond or absconded during current detention (ref: no)	43	6 (13.95)	3 (23.08)	3 (10.00)	0.37	0.06–2.15	.27

* Indicates variable significantly discriminated accepted vs rejected referrals for special security at the State Hospital (N = 149) in Pimm et al. (2004). Ref indicates the reference level for categorical variables.

### Case-control comparisons

Univariate logistic regressions were undertaken to explore differences between accepted and rejected referrals for high-security care. Thirty-six variables were tested, including all eight variables that Pimm et al.^2^ found significantly associated with referral outcome. Results of the univariate analyses are presented in Table 2. There were six statistically significant associations. Accepted referrals were more likely to be individuals who were in prison at the time of referral, slightly older individuals, prescribed antipsychotics at the time of referral, referred for the assessment and treatment of their mental health, and had previous violent convictions. Rejected referrals were more likely to have a history of self-harm. None of the eight variables significant in Pimm et al.^2^ were associated with referral outcome in this sample.

### Multiple logistic regression

The six variables that showed significant association with high-security referral outcome were included in a multivariate logistic regression model. The predictors tested were: referral from prison (2 levels), age (continuous), referred for assessment/treatment of mental health (2 levels), currently prescribed antipsychotic medication (2 levels), history of self-harm (2 levels), and previous violent convictions (2 levels). A reduced sample size of 34 was available for the multiple logistic regression, as complete cases were required.

The parameters retained in the final model are reported in Table 3. Three predictors were retained: Age, currently prescribed antipsychotic medication, and previous violent convictions. The model was significant (*X*^2^(df = 3) = 19.631, *p* < .001) and explained 62.5% of variance in the outcome variable (Nagelkerke *R*^2^ = .625). The Hosmer and Lemeshow Goodness of Fit test was not significant (*X*^2^ (df = 8) = 3.57, *p* = .89), indicating there was good fit between the observed and predicted outcomes. The model correctly classified 82.35% of cases, including 70.00% of rejected referrals and 87.50% of accepted referrals.

**Table 3.** Parameter information for the final multivariate logistic regression model predicting outcome of high security referrals (n = 34).

Predictor	*B*	*SE*	Wald	*p*-value	OR	95% CI
Age (continuous)	0.14	0.08	2.97	.09	1.15	0.98–1.33
Currently prescribed antipsychotic medication (ref: no)	3.47	1.49	5.47	.02	32.18	1.75–590.96
Has previous violent convictions (ref: no)	2.47	1.23	4.01	.045	11.84	1.05–133.12
Constant	−6.87	2.74	6.29	.01	.001	–

## Discussion

This study aimed to describe the primary demographic, clinical and risk characteristics of individuals referred for high-security care, and examine differences between rejected and accepted referrals. It updates the findings of Pimm et al.^2^ and adds to the literature describing referrals to UK high and special security services.^2,13–18^

During the study period at TSH the referral process worked quickly, with a median 12.50 days (IQR 15.75) from referral to decision (13.50 days referral to admission, IQR = 22.50). Accepted patients were also admitted very quickly, in a median of three days after decision (IQR = 6.50). Reflection on figures published for other UK high-security hospitals^13^ suggests that the State Hospital process works efficiently, perhaps due to receiving fewer referrals and/or due to the benefits of referring clinicians and institutions working within the same managed care network, the Forensic Network.

Sixty-seven percent of high-security referrals made to TSH were accepted, up from 38% reported by Pimm et al.^2^ for referrals to TSH made in 1999. However, it should be highlighted that a limitation to Pimm et al.^2^ was that requests for psychiatric report could not be distinguished from referrals for admission, and the sample did contain both types of requests. In comparison, Völlm et al.^13^ reported a 60% acceptance rate at Ashworth Hospital, Williams et al.^14^ 54% at Broadmoor Hospital, and 41% at Rampton Hospital.^15^ The hospital's acceptance rate more closely resembles more recent data from English high security hospitals,^13,14^ nevertheless it is higher. It is possible that awareness and use of existing guidance among referring clinicians in Scotland leads referrers to an appropriate service in the first instance.^8,10^

This study aimed to describe the characteristics of high-security referrals across several patient domains. Our findings generally align with the primary characteristics of high-security referrals previously described. Referrals to UK high-security services tend to be men (only Rampton Hospital provides high-security services to women at this time), aged early 30s,^2,13–15^ and are most often being referred from prison.^13–16^ Of those referred with symptoms of major mental illness, most often this is schizophrenia or schizophrenia spectrum disorders,^2,15^ while secondary diagnoses of substance use disorders are also common.^15^ A large cohort of referrals to English high-security hospitals present with personality disorder; approximately 1/3 in Völlm et al.^13^ and Williams et al.,^14^ and nearly 60% of referrals in Berry et al.^15^ Symptoms of personality disorder were infrequently reported among this sample, reflecting the Scottish approach to managing offenders with personality disorder in prison.^21^ Like Berry et al.^15^ reported for Rampton referrals, a majority of referrals to TSH had previous psychiatric admissions. Also like Berry et al.,^15^ and similar to Pimm et al.,^2^ the majority of our sample had a history of serious physical violence. In this sample, most individuals (56.36%) were referred for assessment and treatment of their mental illness, more than that referred for reasons relating to an index offence (41.82%) or managing behaviour (41.82%). Eighty percent of individuals in this sample were prescribed antipsychotic medication at the time of referral. This suggests that referring clinicians perhaps viewed admission to high security as a means to treat breakthrough psychotic symptoms or to treat individuals who are refusing antipsychotic medication in prison.

A second aim of this study was to compare rejected and accepted referrals, in order to facilitate an understanding of the factors at the crux of decisions about who requires high-security care. We observed significant differences on just six of 36 variables. In this sample, accepted referrals were more likely to be individuals who were in prison at the time of referral, were slightly older, were prescribed antipsychotics at the time of referral, were referred for the assessment and treatment of their mental health, and had previous violent convictions. Rejected referrals were more likely to have a history of self-harm. After subjecting these six variables into a multivariate logistic regression model, the final model was significant and correctly classified 82% of cases with just three variables: age, previous convictions for violent offences, and being prescribed antipsychotic medications at the time of referral.

While none of the eight variables found significant in Pimm et al.^2^ were associated with referral outcome in this sample, the findings still broadly align. Firstly, Pimm et al.^2^ acknowledged that there may have been confounding variables in their data, reflecting the process and documentation practice of the hospital referral system in place in 1999, rather than differences in the characteristics and needs of the individuals referred. Other variables Pimm et al.^2^ found to be significant predictors of referral outcome, including previous admission to TSH, psychotic features at the time of referral, a family history of mental illness, and evidence that psychotic beliefs may have been involved in the index offence or behaviour leading to referral, seem to point to psychosis driven risk of violence as relevant for admission. Pimm et al.^2^ also reported that accepted referrals were more likely to have previous charges or convictions. Berry et al.^15^ similarly reported that co-occurrence of serious mental illness and a history of violence was associated with high security referral acceptance.

The present findings affirm this relationship. In our study, just presenting with symptoms of psychosis did not differentiate referral outcome, but being prescribed antipsychotic medication did, indicating people who are deemed to need high-security are likely a subgroup of people with psychosis who have an incomplete response to antipsychotic medication or refuse their medication. Similarly, in our study, just having a criminal past did not differentiate outcome, but having a *violent* criminal past did, indicating people who need high-security have a risk of violence, and often severe violence. Finally, in this study, accepted referrals were older than rejected referrals. This variable was included in the final model but did not reach statistical significance. Given that age is a smaller effect, it may serve as a proxy in the analysis for the extent of an individual's psychiatric or forensic history.

Reflecting on the present findings and those reported by Pimm et al.,^2^ the factors differentiating accepted and rejected referrals to the State Hospital have changed in terms of degree following the reorganisation of forensic mental health services in Scotland. At the time of Pimm et al.^2^ data collection, the State Hospital provided conditions of special security, effectively including medium and high security. Now, the hospital provides high-security care within a clearly stratified three-tier estate.^8,9,22^ The differentiating factors identified in the present study are therefore more closely related to the need for high security, rather than just higher security. Nevertheless, these findings converge on the conclusion that it is likely both the severity of mental illness and risk of violence that is associated with the need for a higher level of security, and more severe forms of each factor associated with the need for the highest level of security.

### Limitations

Several limitations apply to this study and limit the generalisability of findings to an extent. A sample of 45 consecutive referrals to high security was available for case-control analysis, and there were only 34 complete cases available for the multivariate regression. A longer period of data collection would have provided a larger sample and therefore greater statistical power to detect differences by referral outcome. The study utilised a retrospective design, similar to previous studies,^2,13–15^ though a prospective design may have provided an opportunity to collect data on additional relevant variables, and reduce missing data. Due to the approach to managing personality disorder among offenders in Scotland,^21^ which differs from that in England and Wales, this sample did not have a substantial personality disorder cohort among referrals. For this reason, the findings may be less generalisable to high-security services in England. However, the exclusion of people with a sole diagnosis of personality disorder from the remit of Scottish forensic psychiatric services does align with several European countries in which forensic psychiatric care is only available to individuals with severe psychiatric disorders, such as Ireland, Latvia, Lithuania and Switzerland.^23^

## Conclusions

There are few salient differences across demographic, clinical and risk domains of people accepted for admission to high-security psychiatric care, compared to those rejected for admission. Individuals who were a little older, who were already prescribed antipsychotic medication, and who had history of violent convictions were deemed appropriate for high security. Both the presence of a serious mental illness and risk of violence, appear to be at the crux of decisions about who requires high-security care in a stratified forensic mental health system. Future research could consider examining the differences between accepted and rejected referrals for transfer from high-security care, which would further help to isolate dynamic factors associated with the need for high security.

## Notes

1. In exceptionally urgent cases where the referral was not discussed prior to admission at the PPM, the case would still be noted at the next PPM following admission, facilitating the identification of these cases for the study sample.
